# Fatty Heart*Read before the Buffalo Medical Club, Nov. 24, 1880.

**Published:** 1881-01

**Authors:** H. R. Hopkins


BUFFALO

MEDICAL AND SURGICAL JOURNAL.

VOL. XX.—JANUARY, 1881.—NO. 6.

### Original Communications.

### FATTY HEART.*

BY H. R. HOPKINS, M. D.

*Mr. Chairman and Gentlemen:* I wish to direct your thoughts this evening to the consideration of the fatty diseases of the heart. The subject merits special study for varied reasons. The conditions are most common, cases are met in both sexes, and in all periods of life, from youth to heavy age. Whenever, and in whomsoever occurring, the condition is ever a serious one—one that attacks the patient's health, or even his life; is one in which the natural tendency is not towards a spontaneous and favorable termination.

If my recollection serves me right, our text books do not present this subject in a comprehensive or exhaustive manner; but give it a rather cursory treatment, and the student must find in monographs and the magazines, the literature which will be at all satisfactory.

I esteem it absolutely imperative, that each member of the Medical Club should have much more exact and practical know-

* Read before the Buffalo Medical Club, Nov. 24, 1880.

ledge upon the subject, than is possessed by our average advanced practitioner, and extremely desirable that we should all know more of the matter than is known by the most of our teachers and writers.

It may be that my feeling upon this matter is exaggerated, in which case I have perfect confidence that you will make the neccessary correction, but in three instances, since the subject of this paper was announced, have I seen cases of fatty disease of the heart in the practice of the most eminent men in our local profession, go on to their deaths, without having received one single bit of advice that showed that their medical attendants realized the nature and dangers of the situation.

Surely, if the advice these men received represented the full wisdom of our art, then that art is alarmingly undeveloped.

Another reason why we should study this subject, is from its relation to the causes which determine the premature breakdown and death of so many of our prominent men. Those of you who have lived here for ten, or even for five years, know that the span of life of our active men seldom reaches three score years, and so far as my observations go, fatty disease of some of the organs of circulation, was present as the cause of death more frequently than any single condition.

I trust, therefore, gentlemen, that you all feel, that no matter what I may have to offer you, the subject at least is worthy your most careful consideration.

Out of regard to the tastes of those of you, who delight in exact thinking, I will endeavor to define and limit my subject.

Fatty heart is a clinical term, has no clear pathological meaning, and when used as I propose to use it, should first be explained.

You are aware, that recent authors mention three different kinds of fatty hearts. 1st, Where the heart is externally loaded with an abnormal amount of fat; 2d, The condition of fatty infiltration; and 3d, That of fatty degeneration.

My impression is that the existence of the first of these conditions is little more than problematical, and if it exists as a cause of serious heart failure, the cases are very rare. My reading also convinces me, that it is at present impossible to diagnose between fatty infiltration and fatty degeneration. Therefore, for clinical purposes, fatty infiltration and degeneration, may be associated and included in a single term. Therefore, we will understand by "fatty heart" all or any of those conditions of fatty overgrowth, infiltration or degeneration, which result in an appreciable weakening of the heart's contractile force, and are accompanied by more or less of the subjective signs of heart failure. I do not think it necessary to take up your time, by any extended statement of the pathological condition included under the above titles. The conditions are quite simple and the books agree perfectly regarding the descriptions. I will simply call your attention to a few points in the histology of fatty degeneration. This condition is one in which disease and death attack the primitive elements of the muscles of the heart, is a molecular necrosis of the fibrils, which make up those muscles. The histological elements of the normal heart are very distinct and very beautiful. The fibrillæ show regular and clear cut transverse striæ and nuclei. Fatty degeneration first shows its presence by an indistinctness of the striæ, and a granular appearance of the nuclei. As the disease advances, the granular specks about the nuclei coalesce, and the striæ, from being indistinct, become granular, then are replaced with rows of fat globules. This process of fat formation may go on until the whole substance of a fibril is reduced to a sarcolemma and contains oil globules. This is what the microscope tells us of fatty degeneration. We will understand better what the condition really is, if we examine the process as seen in other muscles.

Anatomists have for a long time known that muscles kept for a time in perfect rest, as in the treatment of a broken bone, were liable to fatty degeneration, and the inference is generally held, that the loss of power in a given muscle is in direct proportion to the presence of the fatty disease. The same condition is also seen in muscles atrophied from loss of motor nerve supply.

The supposition is that in all of these conditions, from varied causes there has been produced a profound impairment of the nutrition of the muscle, which has resulted in its non-renewal. The molecules functionally spent by work, are not replaced by others of more recent formation and fresh powers. The effect upon the functional integrity of the heart, of any extended disease of this nature, can be most easily seen, and it must be the case that the presence of fatty degeneration in a single fibril takes just so much from the working power of the organ; that in just so much as a heart is fatty, that heart is weak; that the clinical history of fatty heart must ever be a history of heart failure.

In this connection I must call your attention to one class of cases, in which the facts would seem to be at variance with the above law. Not an inconsiderable number of cases of fatty heart give a mixed history, and show signs at once of cardiac enlargement and cardiac failure. In the history you will hear an account of conditions that must be accompanied with a powerful and vigorous heart, but recently there have come signs which point to cardiac failure. To these cases writers give the name Failing Hypertrophy, and teach us that hypertrophy is quite frequently succeeded by fatty disease. Consequently it may happen that decided fatty disease may be found in a heart of much more than average power. Nevertheless the law stands as before stated, fatty degeneration is a necrobiotic process, and tends to destroy the functional integrity of the heart.

The consideration of the causes operating to produce fatty heart merits our most earnest study. The gravity and frequency of the disease, together with the powerlessness of our art to alleviate or control its deadly tendencies, makes it our bounden duty to inform ourselves thoroughly as to the causation of the malady.

I will not try your patience by rehearsing generalities regarding what is not known of the causation of fatty heart, nor by reciting all of the varied causes and conditions which, under certain circumstances, are claimed to exert more or less influence in the production of this state, but will limit myself to the presentation of the influence of one or more of the causes of this disease, mainly for the reason that the books do not give them the prominence they would seem to deserve.

First and foremost among the causes of fatty heart I would place persistent high arterial tension, and we will not go far wrong if we believe that high arterial tension, howsoever produced, can not be long maintained without causing fatty heart.

Before considering the rationale of these facts, it will be well to glance for a moment at some of the more prominent causes of high arterial tension or, perhaps, at what might be called the prominent condition which causes such phenomena. Murchison's term, Lithiasis, is adopted by Fothergill as the most significant term, which can be applied to that state of the system, in which the blood is poisoned by retained waste products; and many recent writers agree that lithiasis causes spasm of the arterioles and consequent high arterial tension. The result of persistent high arterial tension is probably first shown upon the arteries themselves, and may be indicated by one word, endarteritis. The weight of authorities incline to the opinion that the high tension causes disease of the intima by reason of increased pressure and consequent increased strain.

From endarteritis follows loss of elasticity and perhaps atheroma, and with these conditions the work of the heart is greatly increased. The labor of supplying the various tissues and organs through rigid, instead of elastic tubes, is not only much greater, but the friction upon the inner surface of the ventricle is to the normal friction of the part, as the impact from a blow of the hand upon a table, is to the same blow upon an air cushion. What is the result of this increased resistance and increased friction of the heart? First, hypertrophy, and then degeneration from fatty disease.

In this consideration we have had to do with general conditions, forces and tendencies, but along with these important local causes are found in operation. The integrity of the blood supply to the heart through its coronary arteries, depends largely upon the elasticity of the aorta.

From the location of these arteries, they depend upon the recoil of the aorta for their supply, and consequently atheroma and rigidity of the aorta, means feeble blood currents in the coronary arteries. More than this, the coronaries are peculiarly susceptible to the influence of high tension, and are particularly liable to atheroma. You can easily see the poor chance of a heart, fed by tortuous and narrowed arteries, which in their turn are served by a diseased inelastic aorta. We have an increased demand for work and a decreased supply of fuel. The result to the individual is premature breakdown, and the autopsy shows fatty heart.

Under this head I must not forget to mention that many cases of anæmia are associated with fatty heart. The relationship to me is most interesting, and is one that deserves careful study, but at present I am unable to give you any rationale as to the mode in which the general condition causes the local disease, but will register the surmise that the future may show the heart disease to be the cause rather than the effect. In speaking some time since of the conditions included under the term fatty heart, I confined myself very closely to the histology of those changes, and neglected to mention the special results they wrought upon the heart. It will be well to complete this statement before going further. I called your attention to the fact that fatty heart must always mean failing heart, and we will also remember that the habit of a heart, which finds itself unequal to the task of forcing its usual volume of blood at regular intervals into the circulation, is towards dilatation, and that all authorities agree in giving fatty degeneration an important place among the causes of dilatation of the heart. As you well know from dilatation comes valvular incompetency, and with incompetent valves and dilated and weakened walls, there is associated a chain of evils, threatening the integrity of every organ and tissue in the whole body. Hyperænia, as of the brain, the lungs, the stomach, the liver, the intestines and the kidneys, with impaired functions of these organs, are due to hearts with incompetent valves and weakened walls. I remember well a case in the care of Dr. Folwell, and I hope the Club will have the pleasure of listening to his report of the same, in which, in the space of eight weeks, life was frequently threatened by secondary affections of most, and I believe of all of the organs above mentioned.

Again, the dilated heart is very liable to rupture, and many cases of fatty heart die suddenly from this cause.

I think there can be no question in any of our minds but that a process so fraught with danger to the heart, and through the destructive changes wrought in the heart so capable of injury to the system at large, is a subject of the highest interest and the gravest importance.

How shall we detect fatty heart? In this, as in many other difficult matters, it is very easy to say how it cannot be done, and I will begin by telling you that you can not detect fatty heart by depending upon physical signs. More than this, that many a member of our profession, more than ordinarily astute in perception and able in judgment, has made a careful physical examination of a patient, whose history pointed to severe disease of some part of the circulatory system, and upon the result of that examination being merely negative, has given the opinion that the cause of the symptoms was elsewhere, that his heart was sound; when the sequel would show that the so-called sound heart must have been rotten with fatty disease.

The literature of the subject plainly teaches that the diagnosis of fatty heart is extremely difficult, in but few cases can it be more than conjectural, that the history of the case is of the utmost importance, and that the question should be approached by an intelligent use of the method by exclusion.

Then there are special difficulties in the way of great variety in conditions and consequently in signs. Fatty heart the successor of large hypertrophy, will vary greatly from the same disease existing in a heart of only average original power. Then the disease is liable to be associated with general plethora and examination of the heart becomes difficult, and in advanced life emphysema frequently co-exists and masks the sounds in a peculiar manner. This is matter of special interest, and I will refer to it again.

My impression is that it is our custom in making an examination of the heart, to try and ascertain as to its size, its regularity of action, its first and second sounds, the absence of abnormal sounds, or if such are present, their seat and nature. As a rule fatty hearts beat regularly, are normal in size, their first and second sounds are clearly heard, and they give no murmurs, Therefore, it is that the average examination of a fatty heart gives only negative results. Experts agree that the peculiarity of the action of the fatty heart consists in a slight modification of the normal sounds, and give prominence to the following.

A diminished apex impulse as felt by the hand, and in a thin person as seen by the eye, and an altered first sound. The exact change in this sound seems difficult to describe, but the testimony in general points in the same direction. One says that this sound is distant and feeble, another that both sounds are far away, less distinct than normal. Many lay stress upon a change in the first sound by which it loses its broad muscular element, which we try to represent by likening the sounds to lub-tip-lub-tip, and retains only its valvular element, and approaches very closely in quality to the second sound. This style of action is not unlike the beating of the fœtal heart, and is represented by the words tick-tick. You will observe that in order to detect these signs we have to pitch our requirements a little higher than merely to determine the two sounds of the heart.

In the above I have had in mind cases of fatty heart presenting the most difficulties of diagnosis. You might say incipient cases, although you must bear in mind that many cases go on to a fatal termination with no more pronounced local signs of disease than those above referred to. In cases where fatty disease succeeds hypertrophy, there will be but slight resemblance to the latter class. Here you will find cardiac enlargement, increased impulse, accentuated second sound, and probably nearly normal first. But in this and where dilatation is present, there is most frequently irregular action, and intermitting pulse. Where dilatation has taken place to the extent of producing valvular incompetency, the heart's action is likely to be rolling, wabbling, tumultous, with murmurs not likely to be harsh in character, but which may be systolic, diastolic, or both. . The gradual development of these abnormal sounds as the heart succumbs to the downward tendencies of the disease, makes most interesting material for clinical study.

You will naturally expect me to say something of the pulse as an indicator of fatty heart.

I have already referred to the intermittent pulse of dilatation and of failing hypertrophy, and in this connection I must tell you one thing about intermittent pulse. There are two kinds of intermittencies. One of no importance and another of the gravest import. The first is where the halt comes along with beats regular as to both time and force, and the other is where the intermission is found associated with want of rythm and unevenness of force of those beats present. All authorities agree that the first may be found in hearts of fully average power and stay, but that the latter is always indicative of advanced degeneration and failure. A limited use of the sphygmograph enables the observer to distinguish, by the finger alone, between these different forms of intermittent pulse, with unerring accuracy.

Hanfield Jones calls attention to the advisability of putting some test upon the heart before estimating its power or integrity; such as a brisk walk about the block or a run up and down a pair of stairs, and then noting its action as compared with the same at rest. Such an experiment often shows that a heart is just capable of doing its work, when that work is reduced to the minimum, but that it has no reserve force—no stay. Aside from its intermittency, the pulse of a fatty heart is always small, feeble, easily compressed and obliterated, and many writers say uniformly rapid. This is frequently one of the first signs of approaching disease, and my observations lead me to consider persistent rapid pulse not congenital, and not otherwise accounted for as a most suggestive symptoms.

True to her love of paradoxes, nature is not content with this, and gives with some marked cases of fatty heart, a notably slow pulse, ranging from 40 to 20 beats per minute. No explanations are given for this contradictory state of things, so far as I know.

This is about all the aid you may expect from physical examination of the heart, and you must remember that there is not one symptom or set of symptoms, that is pathognomonic, or even indicative of fatty heart. They are significant or important when found in connection with a set of subjective signs, which I will try and call to your minds. From what we have seen regarding the remote effects of heart failure, you will not expect me to more than briefly sketch some of the more constant and prominent complaints, which patients suffering from fatty heart are liable to present.

So far as I know, you will first have your attention called to a complaint about the breath, you will hear a story of a quite gradual failure of breathing power, first noticed upon running to catch a car, or to a fire, or upon climbing long flights of stairs, not thought much of for a time, as it passed off at once, but which was more seriously considered when even walking fast, or up a slight incline, or a single flight of stairs, caused its return and it became necessary to sit down to catch breath again. In advanced cases you will hear that the man, from having been one of great endurance, one equal to any effort, has come to be well nigh helpless from want of breath. You satisfy yourself by questioning that this condition of breathlessness has come to be constant upon the slightest exertion. He will also tell you that great muscular weakness has been growing upon him, that if he had as good wind as ever he does not know that he should be able to do much from this trouble.

Then in case you have an opportunity to observe him while sleeping, you will find that his breathing is peculiar, deep and labored respiration alternating with that more and more shallow, until a long pause without breath, to be succeeded by a number of deepening breaths, as if the machinery was running down and getting wound up every 12 to 15 breaths.

But to return to our patient, you have satisfied yourself that he is persistently short-breathed, and he wishes to know the reason why. You can naturally limit your investigation to the lungs, the heart, with a brief glance at the state of the kidneys. You find the lungs are quite normal, perhaps slightly emphysematous, but not in sufficient degree to produce the symptoms. The heart shows no signs of great hypertrophy, gives no murmurs, the vessels give no evidence of aneurism, and the urine is free from albumen. You have finished your examination, your patient sits with eager expectation to hear your verdict, and you have nothing to say. You begin again, and this time a little further back. You ask him his age, and you make a mental note when you hear that he is past fifty. You enquire more particularly regarding his habits, as to eating—how and what, as to drinking, and as to the use of tobacco. You will experience great difficulty in assuring yourself that you have all the facts, but if you are adroit and patient, and perhaps have the assistance of his wife, you finally establish that he has been a large eater, a moderate drinker, and taken but little regular exercise. Then you look at your patient again, a little more closely, you find that he seems older than his years, he is quite gray and bald. From examining his hair you get at his skin, and find that it has an old, smooth, shiny and greasy look, you find that his wife has noticed this, and has repeatedly told him that his skin was like mutton tallow to look and feel. You look at his eyes and find there the true arcus senilis, a ring, partial or entire, about the corneal margin, of yellowish hue with ill-defined outlines. You are careful not to confound this with the calcareous form of arcus, which is complete, is clear and bright with well-defined edges. Upon questioning again, you find that he has had slight attacks of syncope, not severe, but that he could not account for; that his bowels are sluggish, his digestion poor. You must not be surprised if, after you have called to his mind a number of symptoms he had forgotten, to hear him sum up the case with, "I am not the man I used to be, and I guess never will be again.

You must not suppose that the foregoing is intended for an exhaustive, or even a comprehensive sketch of the clinical history of fatty heart. I have seen cases which presented such symptoms, but you will recall that in speaking of the results upon the individual, of the failure of the heart to force the blood through the general circulation at the normal rate and power, I intimated that such failure caused persistent functional and even structural derangements of all of the principal organs and systems of the body, consequently you must be prepared to find a clinical history of the most varied and complicated conditions. After hearing such a history and making such observations regarding the general condition of your patient, you turn again to interrogate the heart, and the general circulatory system. Now, your questioning must be more minute and searching than is the average examiners, if, as the result of your examination, you find the hearts apex impulse feeble, its first sound changed to a distant "tick," its second sound also feeble and distant, the pulse weak, small and easily compressed, then these signs assume decided importance, and justify you in making a diagnosis of fatty heart. In making your examination you must be very careful of the state of the lungs, and of the costal cartilages. A heart beating behind an emphysematous lung and ossified cartilages seems far away and feeble. The neglect of this precaution may lead you to form an erroneous opinion, greatly to the distress of your patient, and ultimately to your own chagrin. Signs of failing hypertrophy or dilatation, such as irregularly intermittent pulse, venous distension, diffused and feeble apex impulse, will aid in bringing your mind to a distinct conception of the case. Are there any affections of the heart likely to be mistaken for fatty degeneration? Smoker's heart, Da Costa's irritable heart and the senile cardiac asthenia of many writers are some of the conditions more frequently brought forward to account for the symptoms of fatty heart. The literature of these conditions plainly shows that for the time; they certainly present an array of symptoms, very like those of fatty heart, and upon one examination, it would be impossible to exclude them. Your ability to do so will depend upon your having a thorough knowledge of the causes, which seem to have operated in bringing about the cardiac failure, and in many cases by observing the progress of the case and the result of treatment.

What shall we say concerning the treatment of fatty heart? In most of your cases your attention will be engaged in the management of the secondary affections of some of the other organs, and you will have but little time or thought to devote to the central and primary disease. You will always remember that a fatty heart is a weak heart, and you will regulate the habits of your patient accordingly. In cases where the disease has not long existed and can be traced to a gouty tendency or to lithiasis, a regimen calculated to correct that habit or condition may reasonably be expected to favorably influence the heart.

When associated with obesity, the management of that tendency by proper diet and exercise will be your aim. When existing with chlorosis or anæmia, this state of the blood will demand attention. For general or special reasons iron, quinine, strychnine, digitalia, belladonna will be ordered and their use countermanded, to give way to other drugs equally useless.

In the great majority of cases, either among the rich or the poor, the diagnosis is not made until the heart is so seriously diseased and disabled, that some secondary trouble seems the most pressing, and engages the attention of the physician. This is one reason why the literature of treatment of fatty heart is so barren and so discouraging. The impression seems to generally prevail that a man with a fatty heart is no better than a dead man. I am persuaded that this is like diagnosing phthisis pulmonalis, only upon the presence of extensive excavations and rating its fatality accordingly. The most marked cases of fatty heart are seen in acute fevers, and yet a large proportion recover; again cases of fatty heart in connection with anæmia are known to recover; again it is known that fatty heart is only produced by the long continued action of its causes, that the heart seems to have remarkable power of resistance, and succumbs at the end of a long struggle. A due consideration of all these facts leads to the conclusion that the earlier diagnosis of fatty heart is necessary, in order that the results of early treatment may be available for intelligent prognosis.

A review of this paper convinces me that my treatment of the subject is not much more satisfactory than the treatment of fatty heart, and at the risk of completely breaking down your tolerance, I will give a brief resume of the points it seemed desirable to emphasize.

1st. The importance of the subject, from its frequency of occurrence, its fertility as a cause of diseased action in other parts, and its universally acknowledged tendency towards a fatal termination.

2d. The necessity of a more thorough knowledge of its causation, in the hope that such knowledge would enable us to do something towards its prevention, as well as furnish the clue for a more successful treatment.

3d. The importance of an intimate and exhaustive knowledge of its clinical history, rendered specially necessary as an assistance in diagnosis.

4th. The need of cultivating the habit of making more careful and minute observations upon the action of the heart, and of improvements in our instrumental aids, particularly those which observe and record the power with which the heart is acting.

5th. That early diagnosis is indispensable to either successful treatment or favorable prognosis.

